# HLA-targeted sequencing reveals the pathogenic role of HLA-B*15:02/HLA-B*13:01 in albendazole-induced liver failure: a case report and a review of the literature

**DOI:** 10.3389/fphar.2023.1288068

**Published:** 2023-11-09

**Authors:** Jin-Mao Liao, Yan Zhan, Zheng Zhang, Jia-Jia Cui, Ji-Ye Yin

**Affiliations:** ^1^ Department of Hepotology, Hunan Provincial People’s Hospital, The First Affiliated Hospital of Hunan Normal University, Changsha, China; ^2^ Department of Clinical Pharmacology, Hunan Key Laboratory of Pharmacogenetics, Xiangya Hospital, Central South University, Changsha, China; ^3^ Institute of Clinical Pharmacology, Central South University, Changsha, China; ^4^ Engineering Research Center of Applied Technology of Pharmacogenomics, Ministry of Education, Central South University, Changsha, China; ^5^ National Clinical Research Center for Geriatric Disorders, Xiangya Hospital, Central South University, Changsha, China; ^6^ Department of Geriatric Surgery, Xiangya Hospital, Central South University, Changsha, China

**Keywords:** IM-ADRs, DILI, HLA polymorphism, genetic susceptibility, liver injury

## Abstract

Drug-induced liver injury (DILI) is one of the serious adverse drug reactions (ADRs), which belongs to immune-mediated adverse drug reactions (IM-ADRs). As an essential health drug, albendazole has rarely been reported to cause serious liver damage. A young man in his 30 s developed severe jaundice, abnormal transaminases, and poor blood coagulation mechanism after taking albendazole, and eventually developed into severe liver failure. The patient was found heterozygous of HLA-B*15:02 and HLA-B*13:01 through HLA-targeted sequencing, which may have a pathogenic role in the disease. This case report summarizes his presentation, treatment, and prognosis. A useful summary of the diagnosis and associated genetic variant information is provided.

## Introduction

Adverse drug reactions (ADRs) are a major problem affecting patient drug use safety (Ribeiro-Vaz et al., 2016; Alfirevic and Pirmohamed, 2017). According to the latest statistics from the World Health Organization, drug-induced damage has risen to the fifth cause of death in the world. The proportion of patients hospitalized due to irrational drug use is 10%–20%, and 5% of the patients died from serious ADRs (Fossouo Tagne et al., 2023). ADRs are divided into predictable type A adverse reactions and unpredictable type B adverse reactions (Bohm et al., 2018). Type B adverse reactions often have more severe consequences, since type B is an immune-mediated adverse drug reactions (IM-ADRs) independent of drug activity (Karnes et al., 2019).

According to the type of immune cells, IM-ADRs can be divided into B cell-mediated IM-ADRs and T cell-mediated IM-ADRs (Yip et al., 2015). The former clinical phenotype develops rapidly and manifests as severe anaphylaxis and mast cell reactions. The latter clinical phenotype is slightly delayed and usually involves vital organs such as the skin, liver, kidney, and pancreas (White et al., 2015), which can lead to the production of many phenotypes, such as Steven-Johnson Syndrome (SJS)/Toxic Epidermal Necrosis (TEN), drug reaction with eosinophilia and systemic symptoms (DRESS), and drug-induced liver injury (DILI). In this study, we focus on DILI, which is an autoimmune disease in the broad sense. DILI can lead to life-threatening liver failure and accounts for 7%–15% of acute liver failure cases in Europe and the United States (Leise et al., 2014). In recent years, many studies have found that DILI shows strong human leukocyte antigen (HLA) associations (such as HLA-B*57:01 and flucloxacillin-induced liver injury (Daly et al., 2009); HLA-DQA1*02:01 and lapatinib-induced liver injury (Spraggs et al., 2011)). HLA is a product encoded by the HLA gene complex. HLA participates in the regulation of the immune system by presenting antigenic peptides to T lymphocytes (Redwood et al., 2018). Here we report a young man with albendazole-induced acute liver failure who carries pathogenic HLA alleles. The role of HLA testing in the diagnosis and prevention of IM-ADRs is highlighted and a review of published similar cases is provided.

## Case description

A young man in his thirties presented to the department of hepatology with a 20-day history of jaundice and abnormal liver function. His mental status was average, with transient appetite loss and poor sleep quality. The disease was exacerbated after admission to the hospital, as the iris and skin of the patient severely yellowed. Additionally, the blood coagulation mechanism deteriorated, and acute liver failure occurred. Before the onset of liver injury, the patient took albendazole tablets orally according to the recommended dose. The patient has a fever after taking the medicine, which was followed by yellow urine and fatigue. He had no preexisting medical conditions. The patient described that he had injured his Achilles tendon playing basketball in 2012 and recovered after surgery. He had two abnormal liver functions due to taking antiparasitic agents in the past. The first time was in childhood, and the drugs used were unknown. The second time was in 2018, and the drug taken was albendazole. Since the condition improved after treatment, the patient did not pay attention to this phenomenon. He had quit smoking for many years but drank alcohol intermittently.

**FIGURE 1:**
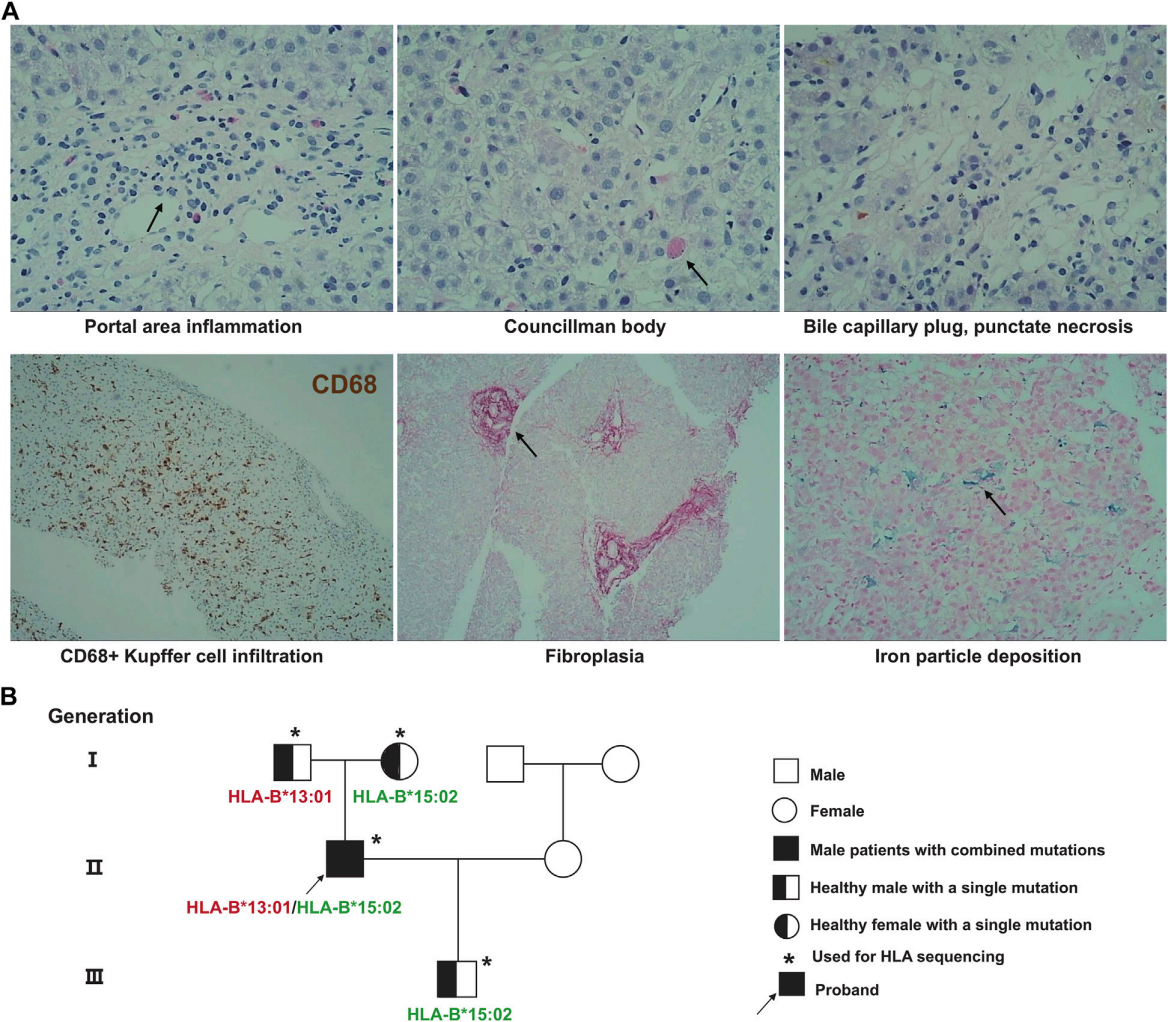
Patient pathological results of liver biopsy and genetic pedigree of HLA polymorphisms. **(A)** The pathological results of liver puncture showed obvious portal area inflammation, eosinophil infiltration, and CD68^+^ Kupffer cell infiltration; **(B)** HLA targeted sequencing results of patients and their families.

The differential diagnosis of unexplained liver injury includes drug-induced liver injury, viral hepatitis, autoimmune liver disease, genetically related liver injury, alcoholic liver disease, parasitic infection, and hepatolithiasis with biliary tract infection. In this case, the patient had no family history of liver disease, and his urine copper and serum ceruloplasmin levels were normal. The patient did not drink alcohol for 1 week before taking albendazole, and also did not have the typical pathological manifestations of alcoholic liver disease. Hereditary liver disease and alcoholic liver disease could be ruled out in advance. Routine blood tests, abdominal B-ultrasound, magnetic resonance imaging (MRI) and magnetic resonance cholangiopancreatography (MRCP) results were normal. Hepatolithiasis combined with biliary tract infection was not considered. A full set of parasite tests was negative, and hepatitis A, B, C, D, E virus, Epstein‒Barr virus, and cytomegalovirus were all negative. A full set of systemic lupus erythematosus, immunity checks, and IgG4 immunoglobulin levels were normal. MRI did not show typical primary sclerosing cholangitis (PSC) bile duct withered branch changes. The patient had a clear medication history and a similar experience after taking albendazole. To make an exclusive diagnosis, a liver biopsy was performed, and the biopsy results supported the diagnosis (Figure 1).

**FIGURE 2:**
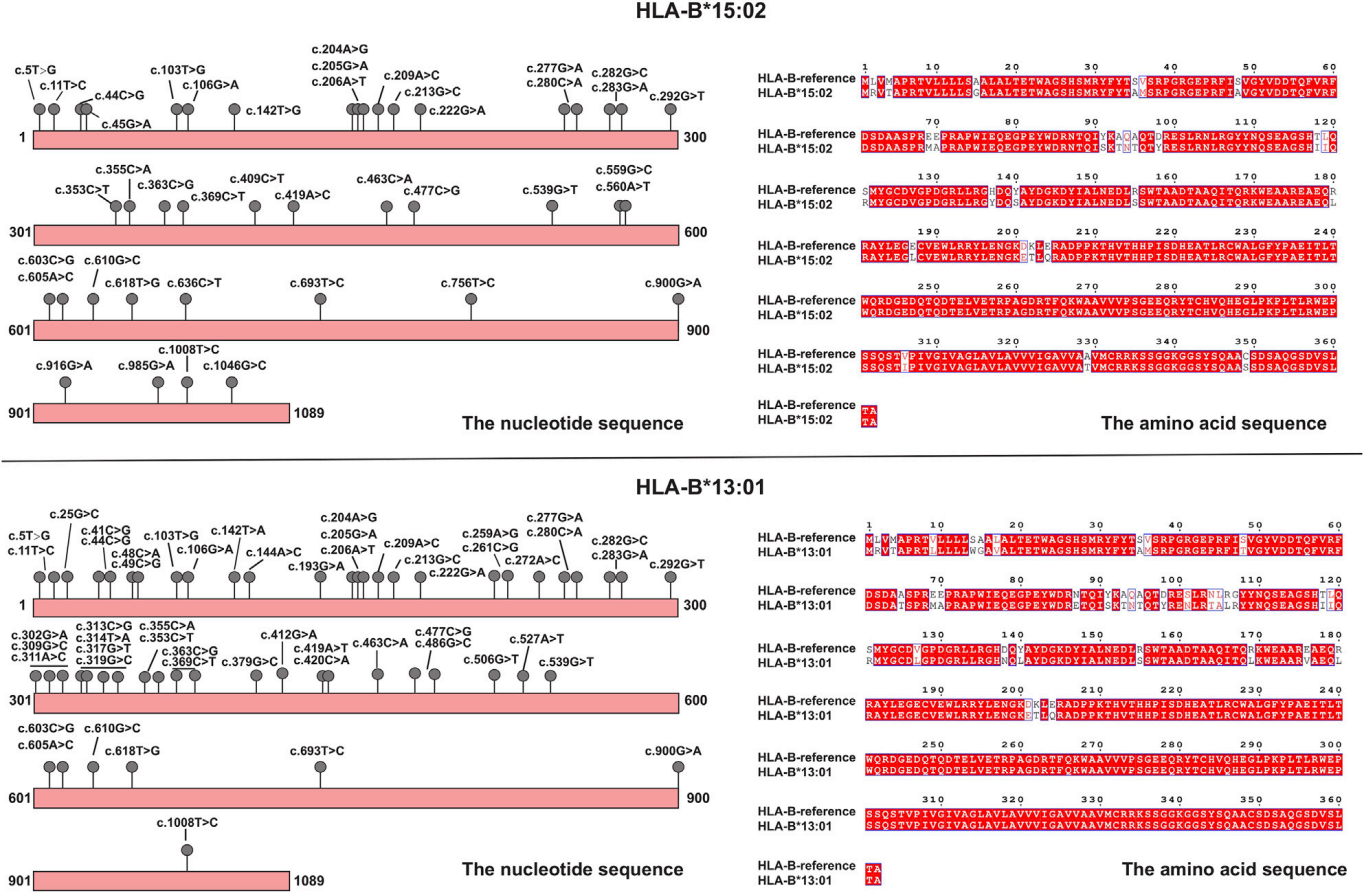
Schematic diagrams of mutations in HLA alleles (HLA-B*15:02 and HLA-B*13:01), and amino acid changes compared to the reference.

Each time the patient took an antiparasitic drug, he developed liver damage of increasing severity. To explore the cause of the disease, we used whole-exome sequencing to detect genetic variants carried by the patient that were consistent with the main clinical phenotype and had clear clinical significance, however, genetic variants that could explain the association with albendazole-induced DILI were not identified. At the same time, we collected the patient’s stool samples for 16s RNA sequencing. The results showed that the gut bacteria levels of patient were within the normal range. The detection values of the nine functional core bacteria, including *Bacteroides*, *Blautia*, *Coprococcus*, *Clostridium*, *Faecalibacterium*, *RoseburiaP*, *Hascolarctobacterium*, *Ruminococcus*, and *Subdoligranulum*, were not deviate from the reference range. We took blood samples from the patient and his family for HLA-targeted sequencing; this technique is a next-generation sequencing (NGS) strategy developed using Illumina sequencing by synthesis (SBS) technology. The sequencing range is the full length of the gene. Genomic DNA for sequencing was extracted from peripheral blood samples, which were performed using the MagPure Fast Blood DNA Kit (Magen Biotech) and quantified using agarose gel electrophoresis. The library construction is completed by NimbleGen kit (Roche Biotech), which can efficiently enrich the human HLA region and its flanks with a total of 4.97 Mb (chr6:28477797–33448354). Fragments between 180 and 280 bp in length were extracted and sequenced using the Illumina NovaSeq6000 system. The sequencing depth was 100x and the coverage was 99%. We found that patient was heterozygous for HLA-B*15:02:01:01 and HLA-B*13:01:01:01. Interestingly, this phenomenon was not observed in any of the patient’s family members, who also took albendazole without developing any adverse effects. Both HLA-B*15:02 and HLA-B*13:01 are alleles of the HLA-B gene. These genotypes consist of serial single-base substitutions. Compared with the HLA-B reference sequence, the HLA-B*15:02 allele has 41 single nucleotide polymorphisms (SNPs) and the HLA-B*13:01 allele has 54 SNPs. Detailed genetic information is shown in Figure 2. These base substitutions cause changes in the amino acid sequence, making the final encoded protein a mutant protein. Studies have shown that mutated HLA proteins can activate the immune system by binding and presenting drugs to T cell receptors, causing adverse drug reactions (Deshpande et al., 2021). Thus, we speculated that these alleles may increase the susceptibility to this disease. The patient not only carries HLA-B*15:02 and HLA-B*13:01, but also has HLA-A*02:03, HLA-A*24:02, HLA-C*03:04, HLA-C*08:01, HLA-DRB1*12:02, HLA-DRB1*16:02, HLA-DQA1*06:01, HLA-DQA1*01:02, HLA-DQB1*05:02, HLA-DQB1*03:01, HLA-DPB1*21:01, HLA-DPB1*05:01, and HLA-DRB3*03:01. Previous studies have found that HLA-DRB1*03:01, HLA-DRB1*04:01 and HLA-DRB1*07:01 are strongly associated with susceptibility to autoimmune hepatitis (de Boer et al., 2014; Terziroli Beretta-Piccoli et al., 2022), and patients with HLA-B8 will accelerate the development of alcoholic cirrhosis (Saunders et al., 1982). However, HLA alleles for AIH susceptibility-associated or accelerated alcoholic hepatitis were not detected in the patient.

After hospitalization, the patient was advised to rest in bed, reduce activities, and eat a high-quality protein diet. Additionally, his vital signs were monitored, and routine blood tests, including liver function, coagulation function, blood ammonia, C-reactive protein (CRP), procalcitonin (PCT), and other indicators, to protect against the development and aggravation of liver failure and coinfection. In terms of drugs, reduced glutathione, magnesium isoglycyrrhizinate injection, and ursodeoxycholic acid capsules were administered. Plasma and cryoprecipitate were intermittently infused to improve blood coagulation function, and energy support therapy was performed. After the patient was discharged from the hospital, liver function was monitored weekly until normalization. During this period, glutathione tablets should be continued for liver protection treatment. Any known drugs to produce ADR related to HLA-B*15:02 and HLA-B*13:01 were excluded from the patient’s medication list, such as carbamazepine and chlorphenylsulfone, to reduce the risk of secondary damage.

The patient gradually recovered liver function through drug treatment, energy support, intermittent infusion of plasma, and coagulation factors. This was accompanied by a decrease in jaundice and an improvement in the coagulation mechanism. After 6 days of symptomatic treatment for jaundice reduction, the patient’s condition improved, all indicators were close to normal, and the skin and sclera had no yellow coloring (Table 1). After being discharged from the hospital, the patient was taking glutathione tablets to protect the liver and rechecked once a week. The half-year follow-up showed that the patient had no symptoms, such as increased jaundice, yellow urine, and fatigue. Enzyme indicators and bilirubin fully recovered 2 months after discharge. In addition, the list of drugs given for the carried HLA alleles also provide clinical references for the patient, and the patient has not experienced liver damage caused by drugs thus far.

**TABLE 1:** Laboratory test results of patients during hospitalization.

Investigation	Results					Reference
	Day 1	Day 4	Day 7	Day 10	Day 14	
Liver function tests						
Total protein	62.37	66.37	64.39	67.13	70.36	65–85 g/L
Albumin	40.02	42.59	41.04	42.21	45.07	35–55 g/L
Globulin	22.35	23.78	23.35	24.72	25.29	20.0–40.0 g/L
Total bilirubin	167.96	303.12	365.12	291.00	125.71	5.1–20.0 μmol/L
Direct bilirubin	141.23	244.67	299.78	242.49	108.10	0–6.10 μmol/L
Indirect bilirubin	26.73	58.45	65.34	48.51	17.61	5.10–20.0 μmol/L
The albumin and globulin ratio	1.79	1.79	1.76	1.72	1.78	1.5–2.5 ng/mL
Alanine aminotransferase	2202.6	1,343.0	608.8	302.6	134.7	9–50 U/L
Aspartate aminotransferase	1,039.8	457.3	178.1	93.7	54.0	15–40 U/L
ALT/AST	2.12	2.94	3.42	3.23	2.49	-
AST/ALT	0.47	0.34	0.29	0.31	0.40	-
Total bile acid	227.48	307.94	201.85	231.26	12.71	0–25.0 μmol/L
**Coagulation test**						
Prothrombin time	-	14.1	13.4	14.1	10.8	9.0–12.5 s
Prothrombin activity	-	59.6	66.9	64.6	89.8	70%–130%
Quantitative fibrinogen	-	2.01	1.67	2.07	2.12	2.00–4.00 g/L
Activated partial thromboplastin time	-	34.0	34.5	34.7	32.3	25.0–34.0 s
Thrombin time	-	23.8	25.3	20.3	19.9	14.0–21.0 s
D-dimer quantification	-	0.34	0.32	0.36	0.37	0–0.55 mg/L
Antithrombin III activity assay	-	81.9	80.0	78.4	97.3	82%–132%
Fibrinogen degradation products	-	1.2	1.5	1.80	1.90	0–5 μg/mL

## Discussion

As a broad-spectrum antiparasitic drug, albendazole can selectively and irreversibly inhibit the aggregation of intestinal parasites and intestinal parietal cell cytoplasmic microtubule system, thereby blocking the uptake of various nutrients and glucose and absorption (Eid et al., 2020). Albendazole has been included in the list of essential medicines by the World Health Organization, and has low toxicity and high efficiency. Reported side effects of the drug include diarrhea, abdominal pain, dizziness, fever, and rash (Bagheri et al., 2004). However, liver injury or acute liver failure rarely occurs, and the pathological mechanism of liver injury is unclear. *In vitro* experiments have demonstrated rapid conversion of albendazole to a sulphoxide (ABS) and subsequently a sulphone (ABSO). ABS is considered an active substance that performs pharmacological effects, while ABSO is an inert compound (Gottschall et al., 1990). The production of ABS in human liver is mediated through flavin monoxygenases (FMO) and cytochrome P450 reductase (CYP), mainly CYP3A4 (Rawden et al., 2000). Therefore, functional alleles on the gene encoding CYP3A4, such as CYP3A4*4, CYP3A4*5, CYP3A4*6, CYP3A4*21 and CYP3A4*22, will affect the efficacy and toxicity of albendazole. ADRs are divided into dose-dependent type A and dose-independent type B. CYP alleles may induce other adverse effects of albendazole (dose-dependent). However, DILI is a dose-independent and unpredictable ADR, it may be less affected by CYP alleles.

To date, a literature search reveals only 10 cases of albendazole-induced liver injury (Table 2), one in which drug-induced liver failure requiring liver transplantation developed (Aasen et al., 2018; Piloiu and Dumitrascu, 2021). According to Table 2, personal history of hepatic parasites is a recurrent indication. Albendazole plays an important role in the treatment of parasitic infections, either as monotherapy or as an adjunct to percutaneous drainage or surgery. Therefore, when using albendazole in the treatment of parasitic infections, clinicians should be aware of the potential for hepatic injury and routinely monitor liver function tests throughout the course of treatment. In addition to a history of parasitic disease, a history of alcohol consumption is an important factor interfering with the diagnosis of drug-induced liver injury. Alcohol is mainly metabolized by the liver, and alcohol consumption increases the burden on the liver and may lead to worsening liver injury. At this time, if the patient carries disease susceptibility-related HLA alleles, the risk of severe liver injury increases exponentially. The description of our patient emphasizes the safe use of albendazole within this context. The patient’s alertness to albendazole is not strong. Safety, high efficiency, and low toxicity have become the stereotype of albendazole, and it is difficult for patients to realize that the onset of symptoms may represent an ADR. Due to the immune memory produced by the body, when exposed to the same stimulus, a stronger immune response will appear, causing more severe liver damage.

**TABLE 2:** A review of case reports of albendazole-induced liver injury.

Authors	Journal/year of publication	Country	Age/Gender	Symptoms	Previous disease/liver biopsy	Mutation testing	Reference
Marin Zuluaga, J. I. et al	*J Med Case Rep*/2013	Colombia	25 years/Female	Pain, fatigue, jaundice	No/Yes	No	Marin Zuluaga et al. (2013)
Nandi, M. et al	*Indian Pediatr/*2013	India	5 years/Male	Fever, vomiting, anorexia, jaundice	No/No	No	Nandi et al. (2013)
Shah, C. et al	*Trop Gastroenterol*/2013	India	7 years/Male	Nausea, anorexia, vomiting, jaundice	No/No	No	Shah et al. (2013)
Ríos, D. et al	*Colomb Med (Cali)*/2013	Colombia	47 years/Male	Jaundice	No/No	No	Rios and Restrepo (2013)
Ben Fredj, N. et al	*Scand J Infect Dis*/2014	Tunisia	35 years/Unknown	Pain, jaundice	Surgery for hydatid cysts 2 years before presentation/No	No	Ben Fredj et al. (2014)
Choi, G. Y. et al	*J Korean Med Sci*/2008	Korea	47 years/Male	Fever, chill, myalgia, vomiting, skin rash	drinking history/No	No	Choi et al. (2008)
Bilgic, Y. et al	*Acta Gastroenterol Belg*/2017	Turkey	47 years/Female	Anorexia, vomiting, jaundice	Cholecystectomy/Yes	No	Bilgic et al. (2017)
Gözüküçük R et al	*Turk J Gastroenterol*/2013	Turkey	28 years/Male	Allergic dermatitis	Surgery for hydatid cyst/No	No	Gozukucuk et al. (2013)
Ben Fredj, N et al	*Scand J Infect Dis*/2014	Romania	22 years/Female	Nausea, vomiting, and headache	Cardiac valvular disease/No	No	Ben Fredj et al. (2014)
Aasen TD et al	Exp *Clin Transplant*/2018	USA	38 years/Female	Worsening malaise, nausea, fatigue, jaundice, acute liver failure	Surgery for hydatid cyst/Yes	No	Aasen et al. (2018)

Recent studies have shown that there is a strong genetic susceptibility to IM-ADRs. And these variants point to the region where the human leukocyte antigen is located (Hetherington et al., 2002; Tassaneeyakul et al., 2009; Kang et al., 2011; Garon et al., 2017). HLA is divided into class I and class II, both of which are distributed on the cell surface and play the role of presenting antigens (Gibson et al., 2023). However, it should be noted that class I HLA molecules present endogenous antigens, while class II responsible for exogenous antigens. The immune response is initiated through the formation of MHC-antigen peptide-TCR complexes (White et al., 2015). Correlative clinical phenotypes include severe cutaneous adverse reactions, DILI, and hypersensitivity syndromes, which affect multiple organs simultaneously. In our study, HLA-targeted sequencing revealed this patient to be heterozygous for HLA-B*15:02 and HLA-B*13:01. HLA-B*15:02 is associated with severe cutaneous adverse reactions of aromatic antiepileptic drugs (AEDs), including phenytoin (Man et al., 2007; Lochar et al., 2008), lamotrigine (Shi et al., 2011), carbamazepine (Chen et al., 2011), and oxcarbazepine (Che et al., 2009). In addition, adverse reactions of some antibiotics (sulfamethoxazole/trimethoprim (Kongpan et al., 2015), and dapsone (Tempark et al., 2017)) were related to HLA-B*15:02. A large clinical trial in Asians revealed a strong association of carbamazepine adverse reactions with HLA-B*15:02 (OR = 1,357, 95% CI = 193–8838, and *p*-value as *p* = 1.6 × 10^−41^) (Chen et al., 2011). The phenotypes involved are Stevens-Johnson syndrome (SJS), toxic epidermal necrolysis (TEN), and drug reactions with eosinophilia and systemic symptoms (DRESS). HLA-B*13:01 was strongly associated with trichloroethylene-induced hypersensitivity dermatitis (OR = 27.5, 95%CI = 3.5–55.7, and *p*-value as *p* = 1.48 × 10^−21^) (Li et al., 2007) and dapsone-induced hypersensitivity reactions (OR = 20.53, 95%CI = 11.55–36.48, and *p*-value as *p* = 6.84 × 10^−25^) (Zhang et al., 2013). The frequencies of HLA-B*15:02 and HLA-B*13:01 in Chinese population are 0.1287 and 0.0680 respectively (Trachtenberg et al., 2007; Yao et al., 2009). However, the patient’s family members carried only one of the two HLA polymorphisms and did not develop DILI after taking the drug. This is an interesting clinical phenomenon, and we highly suspect that HLA alleles play an important role in disease susceptibility.

From the patient’s perspective, the medication recommendations based on their HLA genotype will help to prevent future ADRs.

## Data Availability

The datasets presented in this study can be found in online repositories. The names of the repository/repositories and accession number(s) can be found below: NCBI BioProject (https://www.ncbi.nlm.nih.gov/bioproject/), PRJNA992780.
